# Acceptability of a Pain History Assessment and Education Chatbot (Dolores) Across Age Groups in Populations With Chronic Pain: Development and Pilot Testing

**DOI:** 10.2196/47267

**Published:** 2023-10-06

**Authors:** Nicole Emma Andrews, David Ireland, Pranavie Vijayakumar, Lyza Burvill, Elizabeth Hay, Daria Westerman, Tanya Rose, Mikaela Schlumpf, Jenny Strong, Andrew Claus

**Affiliations:** 1 RECOVER Injury Research Centre The University of Queensland Herston Australia; 2 Tess Cramond Pain and Research Centre The Royal Brisbane and Women's Hospital Metro North Hospital and Health Service Herston Australia; 3 The Occupational Therapy Department The Royal Brisbane and Women's Hospital Metro North Hospital and Health Service Herston Australia; 4 Surgical Treatment and Rehabilitation Service (STARS) Education and Research Alliance The University of Queensland and Metro North Health Herston Australia; 5 Australian eHealth Research Centre The Commonwealth Scientific and Industrial Research Organisation Herston Australia; 6 The Walter and Eliza Hall Institute of Medical Research Melbourne, Victoria Australia; 7 School of Health and Rehabilitation Sciences The University of Queensland St Lucia Australia; 8 Queensland Interdisciplinary Paediatric Persistent Pain Service Queensland Children’s Hospital South Brisbane Australia

**Keywords:** chronic pain, education, neurophysiology, neuroscience, conversation agent, chatbot, age, young adult, adolescence, adolescent, pain, patient education, usability, acceptability, mobile health, mHealth, mobile app, health app, youth, mobile phone

## Abstract

**Background:**

The delivery of education on pain neuroscience and the evidence for different treatment approaches has become a key component of contemporary persistent pain management. Chatbots, or more formally conversation agents, are increasingly being used in health care settings due to their versatility in providing interactive and individualized approaches to both capture and deliver information. Research focused on the acceptability of diverse chatbot formats can assist in developing a better understanding of the educational needs of target populations.

**Objective:**

This study aims to detail the development and initial pilot testing of a multimodality pain education chatbot (Dolores) that can be used across different age groups and investigate whether acceptability and feedback were comparable across age groups following pilot testing.

**Methods:**

Following an initial design phase involving software engineers (n=2) and expert clinicians (n=6), a total of 60 individuals with chronic pain who attended an outpatient clinic at 1 of 2 pain centers in Australia were recruited for pilot testing. The 60 individuals consisted of 20 (33%) adolescents (aged 10-18 years), 20 (33%) young adults (aged 19-35 years), and 20 (33%) adults (aged >35 years) with persistent pain. Participants spent 20 to 30 minutes completing interactive chatbot activities that enabled the Dolores app to gather a pain history and provide education about pain and pain treatments. After the chatbot activities, participants completed a custom-made feedback questionnaire measuring the acceptability constructs pertaining to health education chatbots. To determine the effect of age group on the acceptability ratings and feedback provided, a series of binomial logistic regression models and cumulative odds ordinal logistic regression models with proportional odds were generated.

**Results:**

Overall, acceptability was high for the following constructs: engagement, perceived value, usability, accuracy, responsiveness, adoption intention, esthetics, and overall quality. The effect of age group on all acceptability ratings was small and not statistically significant. An analysis of open-ended question responses revealed that major frustrations with the app were related to Dolores’ speech, which was explored further through a comparative analysis. With respect to providing negative feedback about Dolores’ speech, a logistic regression model showed that the effect of age group was statistically significant (*χ*^2^_2_=11.7; *P*=.003) and explained 27.1% of the variance (Nagelkerke *R*^2^). Adults and young adults were less likely to comment on Dolores’ speech compared with adolescent participants (odds ratio 0.20, 95% CI 0.05-0.84 and odds ratio 0.05, 95% CI 0.01-0.43, respectively). Comments were related to both speech rate (too slow) and quality (unpleasant and robotic).

**Conclusions:**

This study provides support for the acceptability of pain history and education chatbots across different age groups. Chatbot acceptability for adolescent cohorts may be improved by enabling the self-selection of speech characteristics such as rate and personable tone.

## Introduction

### Background

Although pain is commonly thought of as a symptom resulting from injury or disease, chronic or persistent pain is considered a disease in its own right [1]. The disease presents as a complex, common, and costly health problem [2]. The Global Burden of Disease 2016 study found that pain-related diseases, lower back pain, and migraine are the leading causes of disability and disease burden globally [3]. Pain is also the main reason why people seek medical care, with 3 of the top 10 reasons being treatment for osteoarthritis, back pain, or headaches [4]. Chronic pain is common across the life span, with high rates of recurrent headaches and abdominal pain reported in pediatric populations [5].

Most approaches to the management of chronic pain incorporate education about pain neuroscience and the evidence for different clinical interventions such as surgery, pain relief medications, and nonpharmacological approaches [6-8]. Used by both adult and pediatric populations, the education aims to shift one’s conceptualization of pain from that of an indicator of tissue damage or disease to a central nervous system output designed to protect the body [9]. Thus, pain education is a clinical tool that empowers people to self-manage their condition through a better understanding of pain and uptake of evidence-based physical, psychological, and social treatment strategies [10,11].

Recent systematic reviews have revealed that pain education can facilitate the ability to cope with persistent pain by decreasing pain catastrophizing and kinesiophobia [7,11], and when combined with exercise therapy, it can result in greater short-term improvements in pain and disability than exercise alone in adults [7]. However, as most research has focused on educating adults with chronic pain, further empirical investigations with pediatric pain populations are required to effectively tailor and implement pain education [8,12].

To facilitate the processing of complex and novel pain education information in pediatric populations, it has been suggested that multiple modalities may be needed such as video clips and animated mobile phone apps [8]. Mobile phone apps might be particularly useful for digital native adolescents. However, some studies have found low levels of engagement for pediatric pain self-management apps, suggesting that further research is needed to understand how adoption can be enhanced [13,14]. In an adult cohort, allowing people with chronic pain to be able to tell their own story through a comprehensive assessment has been identified as a key component in enhancing the experience of pain education [11].

Chatbots, or more formally conversation agents, are increasingly being used in health care settings to provide education [15]. Chatbots are computer systems that enable interactive 2-way communication through a variety of modalities including videos, images, and written or spoken language [15,16]. The versatility of these communication modalities enhances the potential application of educational chatbots in health care settings across a variety of target populations ranging from young children to older adults [15]. Chatbots are more individualized than leaflets and can be easier to navigate than websites. A survey of 100 physicians acknowledged that although chatbots cannot effectively address the needs of all patients, they can play an important role in supporting and motivating them, resulting in potential cost savings and improved patient outcomes [17].

However, there is a paucity of research on the development, automation, adoption, and acceptability of chatbots from the perspective of patients in the health field [18]. Two available chatbots were evaluated in cohorts consisting of adults with chronic pain, and both evaluations revealed high levels of user acceptability and engagement [19,20]. No known study has used a chatbot in a pediatric pain cohort. There is a need for further research that is focused on the acceptability of diverse chatbot formats, resulting in a better understanding of the needs and preferences of different age groups [15].

### Objectives

The aims of this study were (1) to detail the development of a multimodality pain education chatbot (Dolores); (2) to conduct initial pilot testing with adolescents, young adults, and adults with chronic pain; and (3) to investigate whether acceptability and feedback were comparable across age groups following initial pilot testing. It was hypothesized that acceptability would be comparable across age groups based on a systematic review, indicating that age does not have a consistent effect on health information technology acceptance [21].

## Methods

### Chatbot Development and Framework

Dolores was designed to be embedded in the Pain ROADMAP monitoring app [22,23]. The idea for Dolores resulted from a working group with researchers and clinicians who discussed how to adapt Pain ROADMAP to a pediatric setting. The chatbot framework used to build Dolores is an in-house framework developed at the Commonwealth Scientific and Industrial Research Organisation (CSIRO) in Australia. This framework has been used in other health-related chatbots, including Harlie for autism spectrum disorder and Parkinson disease [24,25] and Edna for genetic counseling [26]. The response engine consists of a case-based reasoner that operates based on the structure (syntax) of human communication. The underlying data structure and response algorithm is a radix tree [24], where the nodes are the possible human-uttered words (or wildcard matching syntax). This data structure provides inherent compression of similar utterances and fast lookup times. A response is produced when the traversal of the radix tree structure finds a *template* response at the end of the utterance.

**Figure 1:**
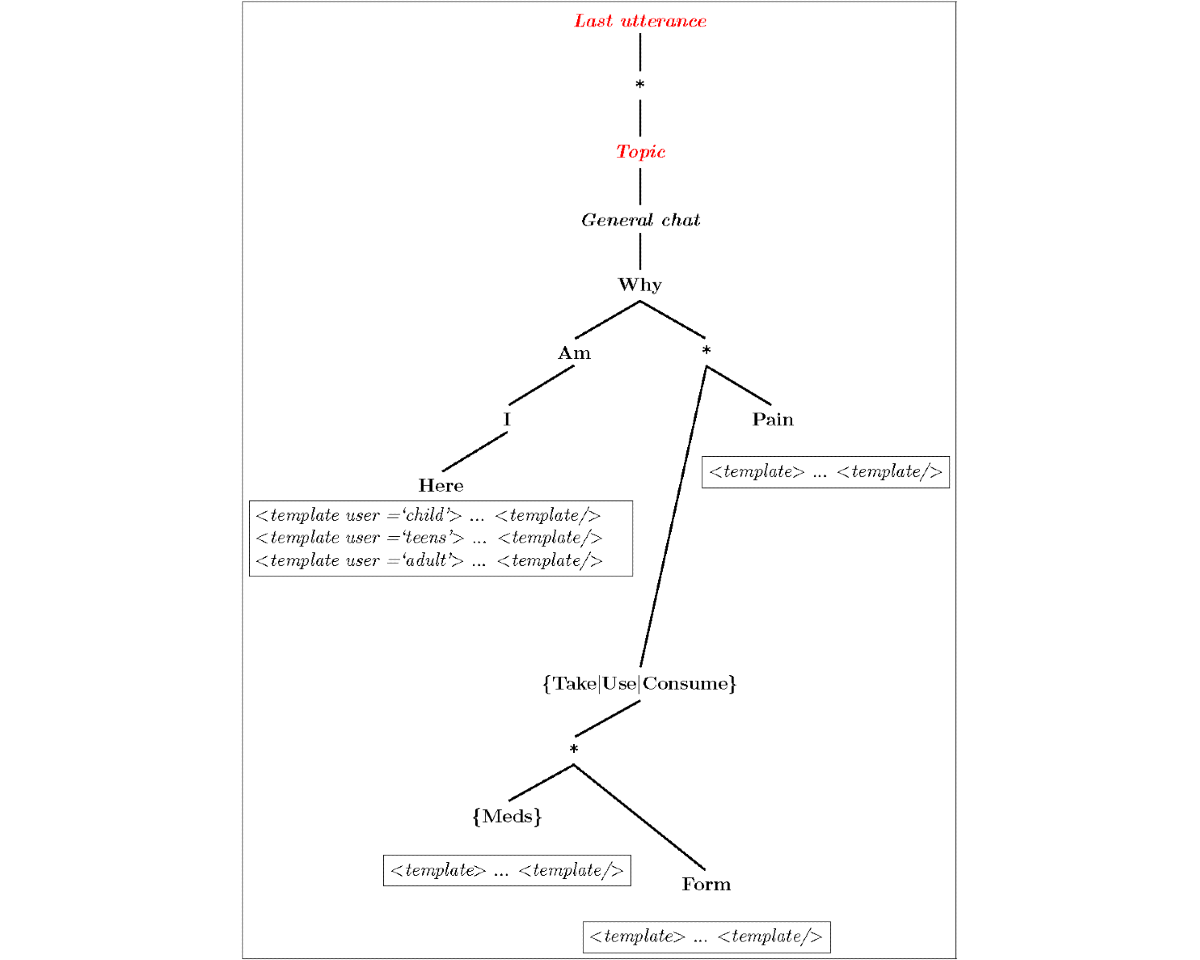
Illustrative example of Dolores’ chatbot radix tree response algorithm developed for pilot testing in a chronic pain population.

Figure 1 provides an example of this process. The radix tree is first created by parsing markup files that contain the patterns (possible human utterances) and corresponding templates that provide instructions on how to respond. The markup language used was based on XML. An example is shown in Textbox 1, which when parsed, forms a part of the tree shown in Figure 1.

The pattern can include wildcards (eg, *: ≥1 words), optional words, sets of words (ie, synonyms), and forbidden words. A constructed radix tree is referred to as a “brain.” The example given in Figure 1 would provide matched responses to utterances such as “Why do I feel pain?” and “Why do I need to take my meds?”

Auxiliary algorithms are also used including sentiment analysis that identifies the probability of negative sentiments and logic reasoning. Multiple responses are encoded, with each specific response chosen based on the sentiment of the last utterance and overall conversation [27]. When Dolores does not have a match in her brain, sentiment analysis is used to determine whether to refer the user to a human or request more information.

**Example of markup language for responding to utterances of the form “Why do I have pain?” “Why do I feel pain?” etc.**

<topic name = “general-chat”>

<category>

<pattern> WHY * PAIN </pattern>

<template>

Pain is a defense mechanism in the brain.

</template>

</category>

</topic>

The design and development of Dolores’ brain was a collaborative process among a group of 6 expert clinicians: 4 occupational therapists, 1 physiotherapist, and 1 speech pathologist who had diverse experiences with clients of different ages, including clinical experience within adult and pediatric tertiary multidisciplinary pain centers (authors NEA, DW, and MS). In CSIRO’s previous chatbot development [24,26], the personality of the person entering the data was evident in the chatbot through subtle language cues; users disengaged quickly unless the programmer who wrote the script was both knowledgeable and personable. Having clinicians write responses for their respected client base provided consistency and portrayed the chatbot as a warm conversation partner with a unique personality.

**Figure 2:**
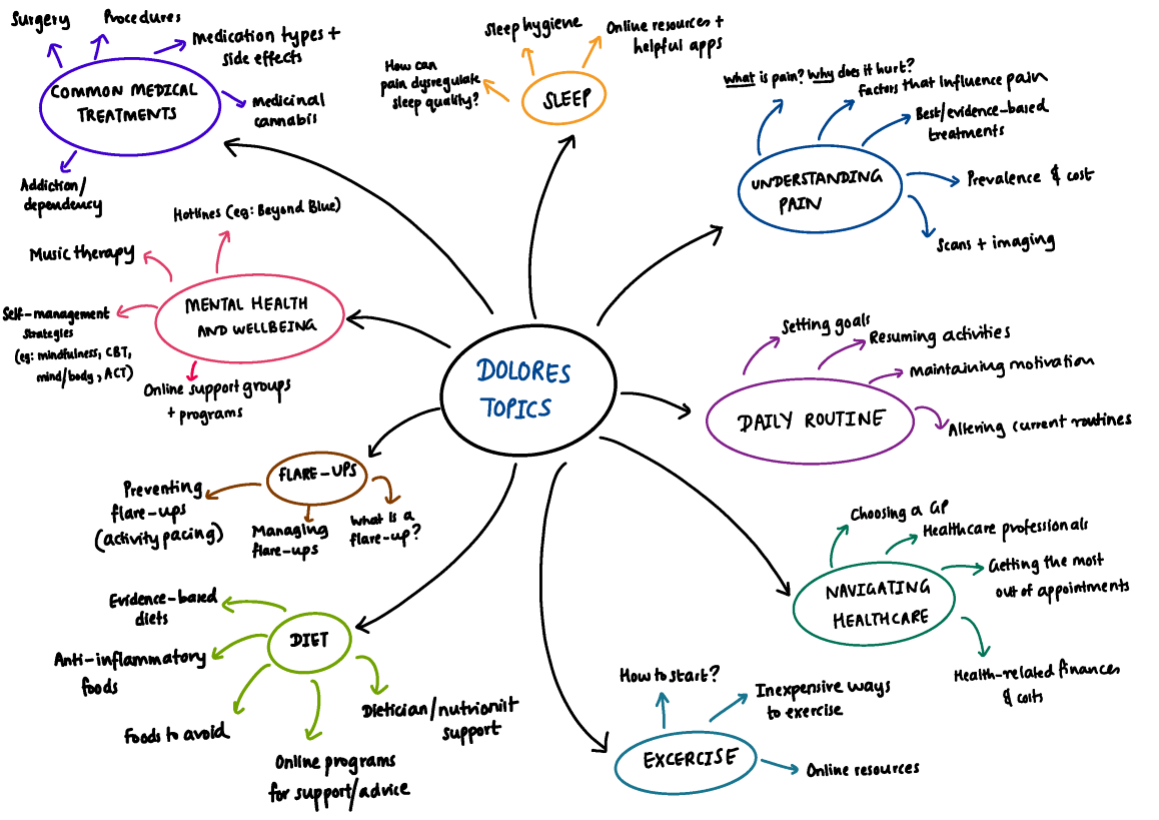
Initial Dolores chatbot education topics that were mapped by the interdisciplinary research team for pilot testing in a chronic pain population.

The initial chatbot education topics were mapped by the interdisciplinary research team with clinical backgrounds in pain management, as illustrated in Figure 2. Commonly asked questions on these topics were then identified, and the subsequent topics, questions, and conversation flows were discussed. First, an adult version of the app content was created. Where possible, existing resources for information were referred to in Dolores’ responses, including web pages, YouTube videos, mobile phone apps, and web-based education modules. This content was then altered to ensure age appropriateness for older and younger adolescents by a pediatric speech pathologist researcher (author TR) and pediatric pain clinicians (authors DW and MS). These alterations included lowering the readability level of the text and referring to more age-appropriate resources. An example of the response to a specific question posed by users of different ages (ie, adults, older adolescents, and younger adolescents) can be seen in Table 1. Implementation proceeded according to the agreed chatbot specifications, and responses were compiled into the brain by an experienced computer scientist (author DI), with assistance from an intern (author PV).

For the purpose of pilot testing, Dolores was built to be used as a stand-alone iOS or iPadOS app. The user interface allowed the clinician to select the language level (ie, adult, older adolescent, or younger adolescent) to match the users’ language proficiency. Dolores generated responses using a combination of speech and written outputs with a “chat bubble” produced as typically seen in a conventional web-based chatting program. The speech output was set to the VoiceOver setting selected on the device. The default setting of Siri Female (Australia) with a pitch change of 50% and speaking rate of 50% was used for pilot testing. An audio recording of the speech output can be found in Multimedia Appendix 1. The user could interact with Dolores through text, speech, and optional interactive buttons. User speech recognition used Google Cloud speech to text, as it has the highest accuracy of available programs and provides a nonlogging option to ensure privacy [28].

On the basis of previous research emphasizing the importance of being able to tell one’s story to enhance the experience of pain education [11], 2 interactive experiences were built. A structured pain history interview was first administered by Dolores, which allowed users to begin developing rapport with Dolores and reflect on their own experiences. Key assessment constructs for the pain history interview were drawn from existing assessment proformas used by local pain centers and the electronic Persistent Pain Outcomes Collaboration (ePPOC) referral questionnaires, which are widely used across Australia and New Zealand [29,30]. The assessment constructs included pain location, pain duration, sensory descriptions of pain, affective descriptors of pain, initial trigger or beginning of the pain experience, perceived impact of pain on daily life, current understanding of pain, and perceptions of treatment approaches. Users could select a drawing widget to answer questions related to pain location, descriptors of pain, and how their pain began. A pain diagram could also be used to mark the location of pain.

**Figure 3:**
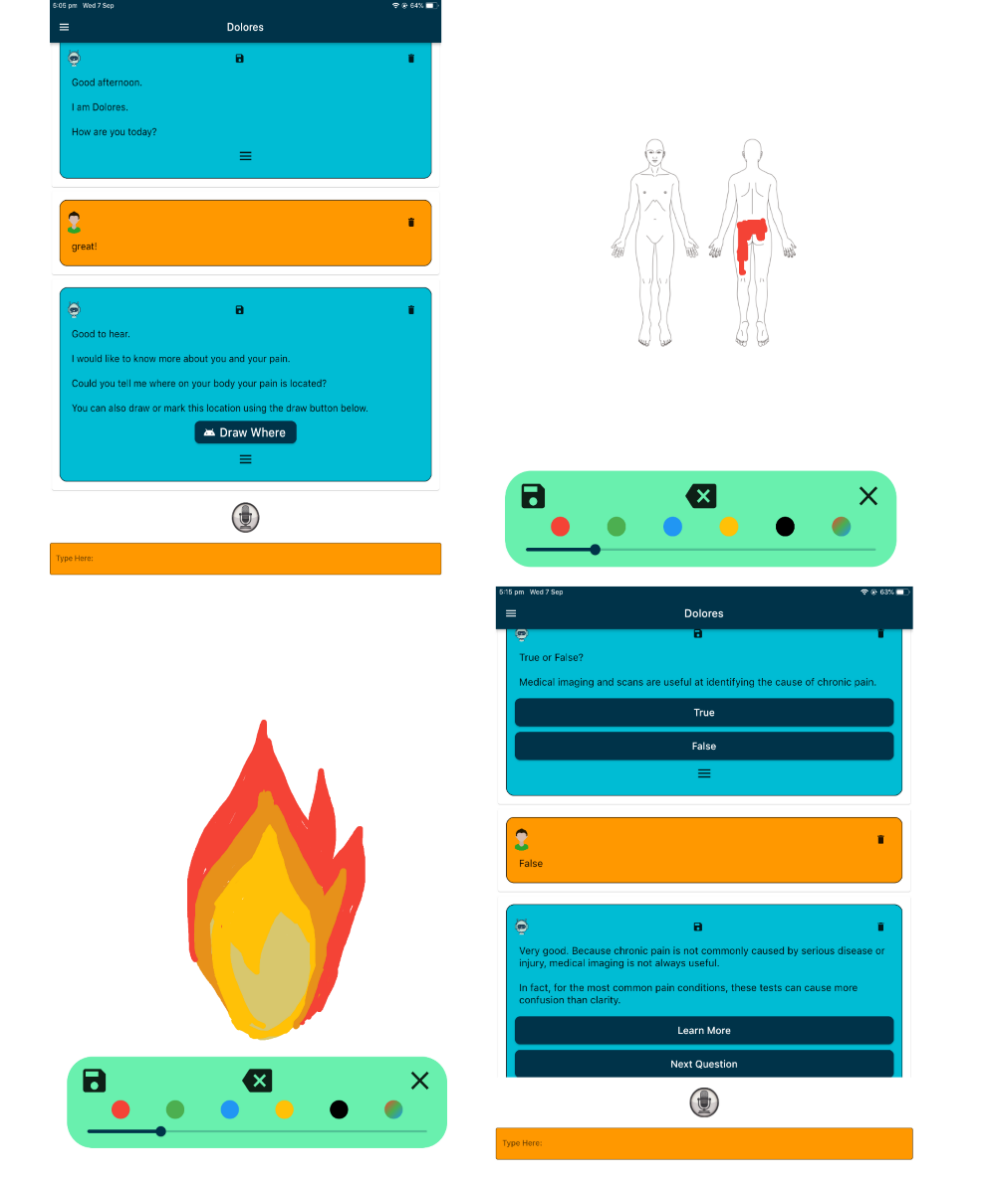
Screenshots of the Dolores chatbot app interface used for pilot testing in a chronic pain population.

The second component of the chatbot interaction is the educational session. Pain education was initiated by Dolores asking the user about their learning preferences. Users were able to browse topics, select a random topic, take a quiz, or ask Dolores questions. If a user wished to continue learning about a topic after the chatbot interaction, the information provided by the Dolores could be saved and then exported to a PDF format for future reference. The chatbot was tested multiple times by the research team before pilot testing with people with chronic pain to verify the appropriateness of the content and conversation flow. Various changes were made to the app layout and content before pilot testing with people with chronic pain (eg, shortening the length of responses and modification of interface labels). Screenshots of the Dolores interface are shown in Figure 3.

**Table 1:** Example of the Dolores chatbot age-appropriate responses to a user’s question that was developed for pilot testing in a chronic pain population.

	Response to the question posed by a user: what is mindfulness?
Response to adults	Mindfulness is about being present in the moment, accepting the thoughts and feelings you’re having without worrying about them, allowing you to achieve a sense of peace. Essentially, mindfulness therapies teach you to be aware of the pain you are feeling and the thoughts you may be having about that pain, without attaching any negative connotations to them. More information about mindfulness can be found at this website [31]. If you want to trial mindfulness, Smiling Mind is a free mindfulness and relaxation app [32]. For more details, visit the Smiling Mind website.
Response to older adolescents	Mindfulness is a skill you can learn to help notice thoughts and feelings in order to feel comfortable and safe. There are many ways to learn mindfulness strategies and your therapist might help you learn how to use this skill as a part of your pain treatment. Talk to your pain team about mindfulness if you would like to know more.
Response to younger adolescents	You can learn how to become more aware of your thoughts and feelings. This is a skill called mindfulness. It can help you to feel relaxed and safe. There are a lot of different things you can do to help you notice what your thoughts and feelings are right now. You might learn how to be more mindful as part of your pain treatment. You can talk to your pain team if you want to know more about mindfulness.

### Ethical Considerations

The Children’s Health Queensland Hospital and Health Service Human Research Ethics Committee (HREC/21/QCHQ/73157), the CSIRO Health and Medical Human Research Ethics Committee (2021_049_RR), and the University of Queensland Human Research Ethics Committee (2021/HE001056) approved the protocol for pilot testing as detailed further. Written informed consent was obtained before participating in the study. For younger participants (aged <18 years), consent was obtained from both the participants and their parents or guardians. All data were deidentified at the time of data collection; participants at each site were allocated a unique research ID number to identify all study materials. All the data were stored on a secure server at the University of Queensland (UQ Research Data Manager). At the end of the study, participants received an Aus $25 (US $18.75) voucher for use in popular retail stores in Australia as a gesture of appreciation. To avoid coercion, participants were not informed of the voucher until the end of the study.

### Pilot Testing With People With Chronic Pain

#### Recruitment and Procedure

Participants were recruited from 1 of 2 pain centers located in large tertiary hospitals in Australia. The inclusion criteria were as follows: the participant must (1) have persistent noncancer pain for at least 3 months; (2) be aged ≥10 years; and (3) possess adequate expressive and receptive language skills to complete the required tasks in English, as determined by clinicians in the treatment team. Participants with coexisting medical conditions were not excluded from this study. As little was known about the variables of interest in this study, the target sample size was based on the rules of thumb for regression (number of predictor variables+50) [33]. A target sample size of 60 participants was used to account for any missing data.

Participants meeting the inclusion criteria were identified by 1 of the 2 physiotherapy research students (authors LB and EH) on presentation to their outpatient appointment. All participants were provided with written and verbal information regarding the study. The research students emphasized that participation was voluntary and that decisions about participation would not affect the patient’s relationship with the clinical staff or their ongoing care at the pain center. Consenting participants were transferred to a private treatment space and completed a short paper-based demographic questionnaire. Participants were then given a fifth-generation iPad with the Dolores app installed. One of the research students provided a 2-minute orientation to the app and provided participants with a cheat sheet (Multimedia Appendix 2). Participants then spent 20 to 30 minutes completing the 2 interactive chatbot activities (ie, the pain history interview and education session). The research student remained in the room during the chatbot interaction to provide technical assistance or answer any questions. However, minimal assistance was required during the pilot testing. On completion of the chatbot experience, participants completed a paper-based custom-made evaluation questionnaire. Adolescent participants were allowed to have their parents or guardians present to assist them in completing all tasks.

#### Measures

##### Demographic Questionnaire

Information regarding participant gender, age, country of birth, languages spoken, language spoken at home, education level, coexisting medical diagnoses affecting communication and language, pain onset, pain duration, and pain location were gathered. Confidence in using mobile phone apps was measured on a 5-point Likert scale (0=not at all confident; 4=very confident). An estimation of the amount of time spent using a smartphone in the last 7 days was also recorded.

##### Custom-Made Evaluation Questionnaire

A custom-made evaluation questionnaire that explored chatbot acceptability was created to ensure that the questions were suitable and easy to understand across all the age groups. Eight acceptability constructs were chosen based on scoping reviews focused on either the technical evaluation of health chatbots or the measurement of mobile health acceptability [34,35]. The 8 constructs examined were engagement, perceived value, usability, accuracy, responsiveness, esthetics, adoption intention, and overall quality. All constructs were rated using a 5-point Likert agreement scale with pictorial representations (ie, emoji faces). Furthermore, 4 open-ended questions were included to allow feedback on the most- and least-liked features and suggestions for future updates. The evaluation questionnaire is presented in Multimedia Appendix 3.

#### Statistical Analysis

All statistical analyses were conducted using SPSS for Windows (version 28.0.1; IBM Corp). Descriptive statistics were first generated to describe the sample and examine feedback pertaining to the Likert-scale acceptability ratings from the evaluation questionnaire. Frequencies were generated for the questionnaire scale responses. A series of cumulative odds ordinal logistic regression models with proportional odds was then generated to determine the effect of age group on acceptability ratings.

Responses to the open-ended questions of the evaluation questionnaire were analyzed using applied thematic analysis with an exploratory approach [36]. Text segmentation was not used, and responses to all the questions were considered in their entirety when generating overarching themes. The text was first analyzed by a researcher with a clinical background in pain management and experience in qualitative analysis (author NA) [37-39] to develop codes and overarching themes. Both codes and themes were reviewed by a second researcher with a background in computer science and experience in qualitative analysis (author DI) [26]. The number of participants whose comments were coded with a particular code (ie, frequency count) was then generated.

The high-frequency codes across the entire sample were examined further. A threshold of a frequency count >10 was used to identify high-frequency codes, and a comparative analysis was undertaken on the high-frequency codes that were distinct from the constructs examined using acceptability Likert scales. Binomial logistic regression models were generated to ascertain the effects of age group on the likelihood of these unique high-frequency codes appearing in the text of open-ended question feedback. An additional binomial logistic regression model was used to establish the effect of age group on providing any comments in the evaluation questionnaire. The following cutoff points were used to interpret the effect size for odds ratios: 1.68 or 0.60 (small), 3.47 or 0.29 (medium), and 6.71 or 0.15 (large) [40].

## Results

A total of 60 individuals with chronic pain were recruited to participate in this study: 20 (33%) adolescent participants (aged 10-18 years), 20 (33%) young adults (aged 19-35 years), and 20 (33%) adults (aged >35 years) with chronic pain.

### Demographic Data

The demographic information pertaining to each age group and the sample as a whole is presented in Table 2. The mean ages of the adolescent, young adult, and adult participants were 15.05 (SD 1.91; range 10-18), 28.79 (SD 4.6; range 21-35) and 54.6 (SD 12.0; range 38-76) years, respectively. More female (14/20, 70%) than male (6/20, 30%) adolescent participants were recruited for the study. An equal number of male and female individuals participated in the other age cohorts. Most participants (54/59, 92%) were born in Australia and identified English as their primary language. The adult group had the highest prevalence of participants (4/20, 20%) who spoke multiple languages. Self-reported coexisting medical conditions known to affect language and communication (eg, dyslexia) were low across the age groups. Overall, the most common pain locations were the lower back (30/59, 51%), upper limbs (25/59, 42%), and neck (25/59, 39%), and medical conditions were the most common cause of pain onset identified (18/59, 31%). The adolescent cohort reported being the most confident in using apps, with 75% (15/20) of participants selecting “very confident.” The time spent using a smartphone was relatively similar across the age groups, with a mean of 25.77 (SD 18.52) hours per week recorded for the sample.

**Table 2:** Demographic information for the chronic pain study cohorts who participated in pilot testing of the Dolores chatbot app.

Demographic variable		Total (n=59)	Adults (n=20)	Young adult (n=19)	Adolescents (n=20)	
**Gender, n (%)**						
	Male	26 (44)	10 (50)	10 (53)		6 (30)
	Female	33 (56)	10 (50)	9 (47)		14 (70)
Age (years), mean (SD; range)		32.88 (18.24; 10-76)	54.6 (12.00; 38-76)	28.79 (4.59; 21-35)	15.05 (1.91; 10-18)	
**Country of birth, n (%)**						
	Australia	54 (92)	16 (80)	19 (100)		19 (95)
	Egypt	1 (2)	1 (5)	0 (0)		0 (0)
	England	1 (2)	1 (5)	0 (0)		0 (0)
	New Zealand	1 (2)	1 (5)	0 (0)		0 (0)
	The Netherlands	1 (2)	1 (5)	0 (0)		0 (0)
	Germany	1 (2)	0 (0)	0 (0)		1 (5)
**Languages spoken, n (%)**						
	English only	54 (92)	16 (80)	19 (100)		19 (95)
	Multiple (including English)	5 (9)	4 (20)	0 (0)		1 (5)
**Main language**, **n (%)**						
	English	59 (98)	19 (95)	19 (100)		20 (100)
	Arabic	1 (2)	1 (5)	0 (0)		0 (0)
**Education level**, **n (%)**						
	University	11 (19)	6 (30)	5 (46)		0 (0)
	Diploma	6 (10)	4 (20)	2 (11))		0 (0)
	Certificate	13 (22)	5 (25)	6 (32)		2 (10)
	Secondary education	20 (34)	5 (25)	6 (32)		9 (45)
	Primary education	9 (15)	0 (0)	0 (0)		9 (45)
**Coexisting medical conditions, n (%)**						
	Dyslexia	3 (5)	1 (5)	0 (0)		2 (10)
	Vision impairment	2 (3)	2 (10)	0 (0)		1 (5)
	Behavior or attention disorder	4 (7)	0 (0)	2 (11)		2 (10)
	Autism spectrum disorder	2 (3)	0 (0)	1 (5)		1 (5)
**Pain onset, n (%)**						
	Injury at home	3 (5)	1 (5)	1 (5)		1 (5)
	Injury at work or school	9 (15)	3 (15)	4 (21)		2 (10)
	Injury in another setting	8 (14)	3 (15)	4 (21)		1 (5)
	Road traffic crash	5 (9)	3 (15)	1 (5)		1 (5)
	Medical condition	18 (31)	8 (40)	5 (26)		5 (25)
	After surgery	5 (9)	1 (5)	2 (11)		2 (10)
	No obvious cause	11 (19)	1 (5)	2 (11)		8 (40)
Pain duration (years), mean (SD; range)		8.09 (8.09; 0.67-40)	15.00 (12.26; 0.67-40)	6.05 (5.51; 1-20.5)	3.12 (3.09; 0.67-14.5)	
**Pain location, n (%)**						
	Head or face	12 (20)	5 (25)	2 (11)		5 (25)
	Lower back	30 (51)	12 (60)	10 (53)		8 (40)
	Upper limb	25 (42)	9 (45)	6 (32)		10 (50)
	Abdomen or groin	15 (25)	4 (20)	4 (21)		7 (35)
	Hips	17 (29)	8 (40)	5 (26)		4 (20)
	Knees	15 (25)	7 (35)	3 (16)		5 (25)
	Neck	23 (39)	10 (50)	5 (26)		8 (40)
	Upper back	16 (27)	3 (15)	6 (32)		7 (35)
	Chest	7 (12)	2 (10)	2 (11)		3 (15)
	Buttocks	4 (7)	3 (15)	1 (5)		0 (0)
	Thighs	7 (12)	3 (15)	2 (11)		2 (10)
	Calves, ankles, or feet	12 (20)	5 (25)	3 (16)		4 (20)
	Total body	2 (3)	1 (5)	1 (5)		0 (0)
**Confidence using apps, n (%)**						
	Not at all confident	0 (0)	0 (0)	0 (0)		0 (0)
	Slightly confident	1 (2)	1 (5)	0 (0)		0 (0)
	Moderately confident	9 (15)	8 (40)	1 (5)		0 (0)
	Confident	20 (34)	8 (40)	36.8 (7)		5 (25)
	Very confident	29 (49)	3 (15)	11 (58)		15 (75)
Hours using smartphone, mean (SD; range)		25.77 (18.52; 0-70)^a^	20.58 (15.13; 2-56)^b^	28.68 (18.88; 3-70)	27.93 (20.84; 0-70)	

^a^One adult nonrespondent, thus n=58.

^b^One adult nonrespondent, thus n=19.

### Likert Scale Acceptability Ratings

The acceptability ratings for the entire sample are presented in Table 3. Overall, the acceptability was high. A very small number of participants (3/57, 5%) indicated that they either disagreed or strongly disagreed with one of the statements. The usability and responsiveness ratings were particularly high, with most participants rating these statements as either agree or strongly agree (54/57, 95% of the sample for usability and 52/57, 91% of the sample for responsiveness). Perceived value was the lowest-rated statement, with 32% (18/57) of the sample recording a neutral response.

The ordinal logistic regression models examining the effect of age group on acceptability ratings are presented in Table 4. The effect of age group on all acceptability ratings was small and not statistically significant. The assumption of proportional odds was met for all models, as assessed by nonsignificant full likelihood ratio tests comparing the fit of the proportional odds model to a model with varying location parameters.

**Table 3:** Acceptability ratings across the whole sample (n=57) based on pilot testing data of the Dolores chatbot app in a chronic pain population.

Acceptability construct	Acceptability Rating					
	Strongly Agree, n (%)	Agree, n (%)	Somewhat Agree, n (%)	Neutral, n (%)	Disagree, n (%)	Strongly Disagree, n (%)
Engagement: I enjoyed talking to Dolores	20 (35)	29 (51)	0 (0)	7 (12)	0 (0)	1 (2)
Usability: It was easy to talk to Dolores	22 (39)	32 (56)	0 (0)	3 (5)	0 (0)	0 (0)
Responsiveness: Dolores was fast enough when responding back to me	30 (53)	22 (39)	0 (0)	5 (9)	0 (0)	0 (0)
Esthetics: I liked the design of the Dolores app including graphics and layout	21 (37)	25 (44)	2 (4)	8 (14)	0 (0)	1 (2)
Perceived value: Dolores helped me to understand more about my pain or my pain treatments	9 (16)	26 (46)	0 (0)	20 (35)	2 (4)	0 (0)
Accuracy: Dolores understood what I was asking or saying	24 (42)	22 (39)	0 (0)	9 (16)	2 (4)	0 (0)
Adoption intention: I would talk to Dolores again	19 (33)	29 (51)	0 (0)	8 (14)	0 (0)	1 (2)
Overall quality: I would recommend Dolores to others	25 (45)	24 (43)	0 (0)	6 (11)	0 (0)	1 (2)

**Table 4:** Logistic Ordinal Regression models investigating acceptability ratings across age groups (n=57) based on pilot testing data of the Dolores chatbot app in a chronic pain population.

Statistic			Engagement		Perceived value		Usability		Accuracy		Responsiveness		Adoption intention	Esthetics		Overall quality
**Pediatric**																
	OR^a^ (95% CI)	2.33 (0.69-7.94)		2.56 (0.76-8.56)		2.68 (0.73-9.78)		1.72 (0.53-5.57)		0.45 (0.27-3.10)		2.67 (0.76-9.19)		1.98 (0.61-6.47)	2.34 (0.69-8.07)	
	*P* value	.17		.13		.14		.37		.90		.12		.26	.17	
**Young adult**																
	OR (95% CI)	1.82 (0.53-6.23)		2.86 (0.84-9.79)		1.15 (0.33-4.02)		1.23 (0.37-4.07)		0.76 (0.22-2.65)		0.88 (0.26-2.98)		0.60 (0.18-2.02)	1.45 (0.42-4.95)	
	*P* value	.34		.09		.83		.74		.67		.84		.41	.55	
**Adult^b^**																
	OR (95% CI)	1.00 (N/A^c^)		1.00 (N/A)		1.00 (N/A)		1.00 (N/A)		1.00 (N/A)		1.00 (N/A)		1.00 (N/A)	1.00 (N/A)	
	Chi-square (*df*)	2.0 (4)		3.6 (4)		2.6 (2)		0.8 (4)		17.1 (2)		3.7 (4)		3.6 (2)	1.9 (4)	
	*P* value	.37		.17		.27		.66		.91		.16		.17	.39	

^a^OR: odds ratio. Higher odds ratios indicate that the age group is more likely to provide negative ratings.

^b^Reference category.

^c^N/A: not applicable.

### Qualitative Feedback

All codes and overarching themes generated from the applied thematic analysis of responses to open-ended questions from the evaluation questionnaire are displayed in Table 5. Most participants (52/57, 91%) provided comments about their experiences by responding to the open-ended questions. The average number of words used to respond to the open-ended questions across the sample was 10.67 (SD 10.22; range 0-56). An analysis of all responses to all questions revealed three overarching themes related to participants’ experiences interacting with Dolores: (1) frustrations, (2) refinements, and (3) benefits.

Most codes relating to the theme *frustrations* concerned Dolores’ speech output; 9 participants expressed that Dolores’ voice was unpleasant or too robotic, 7 participants commented that the speech rate was too slow, and 2 participants highlighted that the pronunciation of certain words was incorrect: “She was a slow talker” (Adolescent participant 12) and “She says words funny” (Adolescent participant 8).

For the theme *refinements*, participant codes encompassed suggestions on how the Dolores app could be improved; 8 participants advocated for adjustable voice options and speech rate settings for Dolores: “a setting to change the speed of talking” (Adolescent participant 11). A further 2 participants recommended an optional mute function for Dolores’ interaction with the users: “have talking be an option” (Adolescent participant 16). Other recommendations that were endorsed by >1 participant included more in-depth or specific information on certain topics, more quiz questions, and the ability to use the app at home.

Under the theme *benefits*, a variety of codes reflected feedback about positive experiences using the Dolores app; 48 participants commented on their positive experiences, whereas 19 participants indicated that the best part of the experience was that the app was easy to use: “easy to use and interact with” (Young Adult participant 2). The efficiency of the app, the layout, and the design were mentioned by 4 participants. The ease with which participants were able to disclose information to Dolores was also highlighted by 4 participants. In addition, 12 participants reported that the information provided by Dolores was useful: “information was brief but helpful” (Young Adult participant 2), whereas 7 participants commented favorably on the multiple options available for user interaction: “I liked the variety of ways to communicate” (Adolescent participant 15).

The different age groups were equally likely to provide feedback by responding to the open-ended questions in the evaluation questionnaire. A binomial logistic regression model examining the effect of age group on providing comments was not statistically significant, and the effect sizes were small (*χ*^2^_2_=0.5; *P*=.78; Nagelkerke *R*^2^=0.02). Dolores’ speech (code A) was considered both a high-frequency code and distinct from the constructs examined using the acceptability Likert scales. The comparative analysis examining the effect of age group on providing negative comments about Dolores’ speech was statistically significant (*χ*^2^_2_=11.7; *P*=.003). The model explained 27.1% (Nagelkerke *R*^2^) of the variance. Adults and young adults were less likely to comment on Dolores’ speech compared with adolescent participants. Using the adolescent cohort as the reference group, a medium effect size was observed for adults (odds 77% lower), and a large effect size was observed for young adults (odds 95% lower).

**Table 5:** Coding and themes from the evaluation questionnaire (n=57) based on pilot testing of the Dolores chatbot app in a chronic pain population.

				Frequency counts^a^, n (%)
**Theme A: frustrations**				19 (33)
	**Code A: Dolores’ speech**			18 (32)
		Code A1: unpleasant voice	9 (16)	
		Code A2: speech rate too slow	7 (12)	
		Code A3: incorrect pronunciation of words	2 (4)	
	Code B: unfamiliar			2 (4)
	Code C: childlike			1 (2)
	Code D: having to type on a touch screen			1 (2)
	Code E: too assertive			1 (2)
**Theme B: refinements**				24 (42)
	Code F: adjustable voice or speech settings			8 (14)
	Code G: more specific or in-depth information			3 (5)
	Code H: more questions in quiz			2 (4)
	Code I: home access or being able to use for longer			2 (4)
	Code J: able to turn off speech option for Dolores			2 (4)
	Code K: add a skip button			1 (2)
	Code L: color codes for pain severity on body map			1 (2)
	Code M: avatar for Dolores			1 (2)
	Code N: more questions in pain history			1 (2)
	Code O: more links to existing resources			1 (2)
	Code P: edit button for typed entries			1 (2)
	Code Q: improved speech to text			1 (2)
	Code R: Bluetooth keyboard			1 (2)
	Code S: easier to erase when drawing			1 (2)
	Code T: monthly tracking of progress			1 (2)
	Code U: additional games related to topics			1 (2)
**Theme C: benefits**				48 (84)
	Code V: easy to use			19 (33)
	**Code W: information useful**			12 (21)
		Code W1: informative	10 (18)	
		Code W2: helpful information	1 (2)	
		Code W3: information had not been provided by health professionals	1 (2)	
	**Code X: options for user to interact, including drawing**			7 (12)
		Code X1: interactive	2 (4)	
		Code X2: multiple ways to respond	3 (5)	
		Code X3: being able to draw responses	2 (4)	
		Code X4: being able to speak to app	1 (2)	
	Code Y: quick or efficient			4 (7)
	Code Z: easy to open up about issues or talk to			4 (7)
	Code AA: good layout or design			4 (7)
	Code AB: good tool or generalized app			3 (5)
	Code AC: mobile or easy to access			1 (2)
	Code AD: asked questions that may not think of			1 (2)
	Code AE: real-time response or feedback			1 (2)
	Code AF: interesting quiz			1 (2)
	Code AG: lets user make mistakes			1 (2)
	Code AH: speech to text accuracy			1 (2)

^a^Number of participants whose comments were coded with a particular code or theme.

## Discussion

### Principal Findings

The utility of chatbots in the health care sector is an emerging field of research. This study details the development and initial pilot testing of a multimodality pain history assessment and pain education chatbot (Dolores) designed to be used across different age groups from early adolescence to adulthood. We hypothesized that the acceptability ratings and feedback would be comparable across different age groups following initial pilot testing.

The overall acceptability ratings were high across the 3 age groups examined (ie, adolescents, young adults, and adults), and the effect of age group on all acceptability ratings was small and not statistically significant. The positive feedback provided to the open-ended questions also outweighed the negative or constructive feedback. The participants provided positive feedback on the multiple options available for interaction and commented that the app was easy to use. Dolores was designed to provide age-appropriate responses in terms of both content and readability level using 3 settings (ie, for younger adolescents, older adolescents, and adults). Dolores was also programmed to accommodate different user communication needs (eg, visual, speech, and text) and learning preferences (eg, taking a quiz, browsing topics, and asking questions). The current settings and options appeared appropriate, as only one participant provided negative feedback regarding the app not being targeted to the participants’ age group (ie, an adult stating that the app was a bit childlike). This is in contrast to a previous study that has revealed that adolescents often feel that health apps are designed for adults, impacting adoption intention [41]. The results of this study suggest that multimodality pain education chatbots are suitable for use across the different age groups examined.

Although most feedback was positive, 30% (18/57) of participants made specific negative comments about Dolores’ speech, which was set to Siri Female (Australia) with a pitch change of 50% and a speaking rate of 50% (Multimedia Appendix 1). Participants reported that the speech rate was too slow, the voice output was unpleasant, or Dolores pronounced words incorrectly. Adults and young adults were statistically less likely to comment on Dolores’ speech compared with adolescent participants. However, the reason for this finding remains unclear. As all age groups were equally likely to provide comments in response to the open-ended questions, the results could not be adequately explained by response bias.

One possible explanation for age-related differences in opinions pertaining to Dolores’ speech may be related to human-robot age congruity. Previous experimental research within the travel industry has demonstrated that human-robot gender congruity elicits more positive affect whereby participants are more likely to rate their level of comfort in the service encounter higher if the robot is perceived to be of the same gender as the participant [42]. Similarly, in the retail industry, research has demonstrated that matching consumer personality with congruent chatbot personality has a positive impact on both consumer engagement with chatbots and purchasing outcomes [43]. As the voice selected for pilot testing resembled an adult’s voice, it may be that adolescent participants were more likely to comment on Dolores’ speech due to age-related incongruencies between the participant’s own voice and Dolores’.

Alternatively, the desired level of anthropomorphism may explain the association between age and negative comments about Dolores’ speech. A narrative review of literature comparing embodied with disembodied chatbots, used in a variety of research fields, yielded contradictory results regarding the effect of human likeness on user acceptability [44]. Current research suggests that extreme anthropomorphic features may lead to cognitive dissonance, whereas a chatbot with no human identity could affect perceptions of trust and the quality of the interaction [16]. However, research examining preferences within pediatric and adolescent cohorts is lacking. It is possible that there is a preference for more fictional personas, such as animals or mythical creatures, within this age cohort. The Kids and Family Reading Report [45], which sampled 2758 parents and their children, revealed that children and adolescents aged 6 to 17 years are equally likely to learn life lessons from fictional and nonfictional characters [45]. The types of characters children and adolescents want in books reflect their own desired personal attributes such as resilience, intelligence, bravery, and strength [45]. No known research has explored the impact of using different chatbot personas with adolescents or children in the health field.

It is unclear how negative perceptions of a chatbot’s speech might impact other acceptability constructs; there were no age-related differences in the acceptability ratings in this study for the construct engagement, perceived value, usability, accuracy, responsiveness, adoption intention, esthetics, and overall quality. It is possible that negative perceptions of chatbots’ speech and persona might impact perceived trustworthiness and long-term engagement, which were not measured. In clinical settings, improved treatment outcomes have been linked to the strength of the therapeutic alliance between therapists and people seeking treatment for chronic pain [46], and perceived trustworthiness has been shown to be important in therapeutic encounters [47]. Participants recommended several minor adaptations to the Dolores app, including mute options and adjustable voice and speech settings. Although these adaptations may improve acceptability, further research is needed to examine chatbot personality characteristics that can optimize trustworthiness and long-term engagement in adolescents.

Incorporating technology into health care for young people is becoming increasingly recognized as an avenue for enhancing care [48,49]. Long-term engagement with chatbots has the potential to increase the ways young people can interact with their health care and allow young people to exercise more control over decisions related to their health [50,51]. Prior research has found that young people aged <17 years exhibit a mean of only 15% of all utterances during a specialist medical appointment and that active participation is increased when young people are engaged directly through a range of strategies that allow them to speak about their daily life, make choices, and indicate their preferences [52]. Chatbots may be highly valuable tools for increasing engagement, communication, understanding, and clinical outcomes in adolescents. Therefore, further research on long-term chatbot use as an adjunct to existing evidence-based interventions and services is warranted.

### Limitations

The results of this study should be interpreted with consideration of the following limitations. The sample size was small**,** and only 2 clinical sites were used to recruit participants, limiting the generalizability of the findings. A custom-made evaluation questionnaire was created to ensure that the measurement was suitable and easy to understand across all age groups. However, this may have introduced measurement errors that affected the confidence of the findings. A researcher was present during the pilot testing to assist with any technical issues. This may have affected chatbot engagement and acceptability due to the Hawthorne effect. Participants were, however, explicitly told that the researchers were only present to provide technical support**,** and they were positioned out of direct sight to decrease the influence of their presence on the participants’ behavior. Broad categorizations were used for age grouping to facilitate recruitment; however, definitions for “adolescent” and “young adult” do vary. Children (ie, aged <10 years) and an older adult group (eg, exclusively those aged >65 years) were not recruited due to perceived difficulties in recruiting these cohorts at the clinical sites participating in the study. All the participants in this study indicated that they were at least slightly confident when using smartphone apps. Younger children or older adults may not have had any exposure to apps, with the potential to reduce acceptability in those age groups. Investigating the acceptability of pain education chatbots with older adults, children aged <10 years, and populations with lower health and digital literacy skills is an avenue for future research.

### Conclusions

This study detailed the development and pilot testing of a multimodality chatbot within the pain field. The study provided support for the acceptability of pain education chatbots for conducting a pain history assessment and delivering education across age groups spanning from early adolescence to adulthood. Adolescent participants were more likely to provide specific negative comments on the chatbot’s speech compared with adults and younger adults. Although chatbot acceptability may be improved by enabling the self-selection of voice and speech settings, more research is needed to understand how the embodiment of chatbots can be optimized, with consideration of audiovisual preferences relating to age and gender, to facilitate long-term engagement as a complement to standard care.

## Appendix

Multimedia Appendix 1 Audio sample of Dolores’ speech from pilot testing the Dolores chatbot app in a chronic pain population.

Multimedia Appendix 2 Orientation to the Dolores app: cheat sheet that was used for pilot testing in a chronic pain population.

Multimedia Appendix 3 A custom-made evaluation questionnaire assessing acceptability constructs relevant to chatbots that was used for the pilot testing of the Dolores chatbot app in a chronic pain population.

## Abbreviations

CSIRO: Commonwealth Scientific and Industrial Research Organisation
ePPOC: electronic Persistent Pain Outcomes Collaboration

## Data Availability

The data sets generated and analyzed during this study are available from the corresponding author on reasonable request.

## References

[1] Treede R-D, Rief W, Barke A, Aziz Q, Bennett MI, Benoliel R, Cohen M, Evers S, Finnerup NB, First MB, Giamberardino MA, Kaasa S, Korwisi B, Kosek E, Lavand'homme P, Nicholas M, Perrot S, Scholz J, Schug S, Smith BH, Svensson P, Vlaeyen JW, Wang S-J (2019). Chronic pain as a symptom or a disease: the IASP Classification of Chronic Pain for the International Classification of Diseases (ICD-11). Pain.

[2] Cohen SP, Vase L, Hooten WM (2021). Chronic pain: an update on burden, best practices, and new advances. Lancet.

[3] GBD 2016 Disease and Injury Incidence and Prevalence Collaborators (2017). Global, regional, and national incidence, prevalence, and years lived with disability for 328 diseases and injuries for 195 countries, 1990-2016: a systematic analysis for the Global Burden of Disease Study 2016. Lancet.

[4] St Sauver JL, Warner DO, Yawn BP, Jacobson DJ, McGree ME, Pankratz JJ, Melton LJ, Roger VL, Ebbert JO, Rocca WA (2013). Why patients visit their doctors: assessing the most prevalent conditions in a defined American population. Mayo Clin Proc.

[5] King S, Chambers CT, Huguet A, MacNevin RC, McGrath PJ, Parker L, MacDonald AJ (2011). The epidemiology of chronic pain in children and adolescents revisited: a systematic review. Pain.

[6] Sharpe L, Jones E, Ashton-James CE, Nicholas MK, Refshauge K (2020). Necessary components of psychological treatment in pain management programs: a Delphi study. Eur J Pain.

[7] Siddall B, Ram A, Jones MD, Booth J, Perriman D, Summers SJ (2022). Short-term impact of combining pain neuroscience education with exercise for chronic musculoskeletal pain: a systematic review and meta-analysis. Pain.

[8] Robins H, Perron V, Heathcote LC, Simons LE (2016). Pain neuroscience education: state of the art and application in pediatrics. Children (Basel).

[9] Moseley GL, Butler DS (2015). Fifteen years of explaining pain: the past, present, and future. J Pain.

[10] Koechlin H, Locher C, Prchal A (2020). Talking to children and families about chronic pain: the importance of pain education-an introduction for pediatricians and other health care providers. Children (Basel).

[11] Watson JA, Ryan CG, Cooper L, Ellington D, Whittle R, Lavender M, Dixon J, Atkinson G, Cooper K, Martin DJ (2019). Pain neuroscience education for adults with chronic musculoskeletal pain: a mixed-methods systematic review and meta-analysis. J Pain.

[12] Ickmans K, Rheel E, Rezende J, Reis FJ (2022). Spreading the word: pediatric pain education from treatment to prevention. Arch Physiother.

[13] Grasaas E, Helseth S, Fegran L, Stinson J, Småstuen M, Lalloo C, Haraldstad K (2022). App-based intervention among adolescents with persistent pain: a pilot feasibility randomized controlled trial. Pilot Feasibility Stud.

[14] Palermo TM, de la Vega R, Murray C, Law E, Zhou C (2020). A digital health psychological intervention (WebMAP Mobile) for children and adolescents with chronic pain: results of a hybrid effectiveness-implementation stepped-wedge cluster randomized trial. Pain.

[15] Tudor Car L, Dhinagaran DA, Kyaw BM, Kowatsch T, Joty S, Theng Y-L, Atun R (2020). Conversational agents in health care: scoping review and conceptual analysis. J Med Internet Res.

[16] Schachner T, Keller R, V Wangenheim F (2020). Artificial intelligence-based conversational agents for chronic conditions: systematic literature review. J Med Internet Res.

[17] Palanica A, Flaschner P, Thommandram A, Li M, Fossat Y (2019). Physicians' perceptions of chatbots in health care: cross-sectional web-based survey. J Med Internet Res.

[18] Parmar P, Ryu J, Pandya S, Sedoc J, Agarwal S (2022). Health-focused conversational agents in person-centered care: a review of apps. NPJ Digit Med.

[19] Sinha C, Cheng AL, Kadaba M (2022). Adherence and engagement with a cognitive behavioral therapy-based conversational agent (Wysa for chronic pain) among adults with chronic pain: survival analysis. JMIR Form Res.

[20] Hauser-Ulrich S, Künzli H, Meier-Peterhans D, Kowatsch T (2020). A smartphone-based health care chatbot to promote self-management of chronic pain (SELMA): pilot randomized controlled trial. JMIR Mhealth Uhealth.

[21] Or CK, Karsh B-T (2009). A systematic review of patient acceptance of consumer health information technology. J Am Med Inform Assoc.

[22] Ireland D, Andrews N (2019). Pain ROADMAP: a mobile platform to support activity pacing for chronic pain. Stud Health Technol Inform.

[23] Andrews NE, Ireland D, Deen M, Varnfield M (2023). Clinical utility of a mHealth assisted intervention for activity modulation in chronic pain: the pilot implementation of pain ROADMAP. Eur J Pain.

[24] Cooper A, Ireland D (2018). Designing a chat-bot for non-verbal children on the autism spectrum. Stud Health Technol Inform.

[25] Ireland D, Atay C, Liddle J, Bradford D, Lee H, Rushin O, Mullins T, Angus D, Wiles J, McBride S, Vogel A (2016). Hello Harlie: enabling speech monitoring through chat-bot conversations. Stud Health Technol Inform.

[26] Ireland D, Bradford D, Szepe E, Lynch E, Martyn M, Hansen D, Gaff C (2021). Introducing Edna: a trainee chatbot designed to support communication about additional (secondary) genomic findings. Patient Educ Couns.

[27] Ireland D, Hassanzadeh H, Tran SN (2018). Sentimental analysis for AIML-based e-health conversational agents. Proceedings of the Neural Information Processing 25th International Conference.

[28] Speech-To-Text. Google.

[29] Tardif H, Arnold C, Hayes C, Eagar K (2017). Establishment of the Australasian electronic persistent pain outcomes collaboration. Pain Med.

[30] Lord SM, Tardif HP, Kepreotes EA, Blanchard M, Eagar K (2019). The Paediatric electronic Persistent Pain Outcomes Collaboration (PaedePPOC): establishment of a binational system for benchmarking children's persistent pain services. Pain.

[31] D'arcy-Sharpe AM (2020). Mindfulness for chronic pain: a comprehensive guide. Pathways Health Ltd.

[32] (2023). Smiling Mind.

[33] Wilson Van Voorhis CR, Morgan BL (2007). Understanding power and rules of thumb for determining sample sizes. Tutor Quant Methods Psychol.

[34] Abd-Alrazaq A, Safi Z, Alajlani M, Warren J, Househ M, Denecke K (2020). Technical metrics used to evaluate health care chatbots: scoping review. J Med Internet Res.

[35] Nadal C, Sas C, Doherty G (2020). Technology acceptance in mobile health: scoping review of definitions, models, and measurement. J Med Internet Res.

[36] Guest G, MacQueen KM, Namey EE (2012). Applied Thematic Analysis.

[37] Andrews NE, Strong J, Meredith PJ, Gordon K, Bagraith KS (2015). "It's very hard to change yourself": an exploration of overactivity in people with chronic pain using interpretative phenomenological analysis. Pain.

[38] Johnston V, Brakenridge C, Valiant D, Ling CL, Andrews N, Gane EM, Turner B, Kendall M, Quinn R (2022). Using framework analysis to understand multiple stakeholders’ views of vocational rehabilitation following acquired brain injury. Brain Impair.

[39] Vaezipour A, Andrews N, Oviedo-Trespalacios O, Amershi F, Horswill M, Johnston V, Delhomme P (2022). Exploring driving behaviour from the perspectives of individuals with chronic pain and health professionals. Appl Ergon.

[40] Chen H, Cohen P, Chen S (2010). How big is a big odds ratio? Interpreting the magnitudes of odds ratios in epidemiological studies. Commun Stat Simul Comput.

[41] Chan A, Kow R, Cheng JK (2017). Adolescents’ perceptions on smartphone applications (Apps) for health management. J Mobile Tecnol Med.

[42] Pitardi V, Bartikowski B, Osburg V-S, Yoganathan V (2023). Effects of gender congruity in human-robot service interactions: the moderating role of masculinity. Int J Inf Manage.

[43] Shumanov M, Johnson L (2021). Making conversations with chatbots more personalized. Comput Hum Behav.

[44] Chaves AP, Gerosa MA (2020). How should my chatbot interact? A survey on social characteristics in human–chatbot interaction design. Int J Hum Comput Interact.

[45] (2019). Kids and family reading report™. 7th edition. Finding their story. Scholastic Inc.

[46] Kinney M, Seider J, Beaty AF, Coughlin K, Dyal M, Clewley D (2020). The impact of therapeutic alliance in physical therapy for chronic musculoskeletal pain: a systematic review of the literature. Physiother Theory Pract.

[47] Buchman DZ, Ho A, Illes J (2016). You present like a drug addict: patient and clinician perspectives on trust and trustworthiness in chronic pain management. Pain Med.

[48] Irwin CE Jr (2020). Using technology to improve the health and well-being of adolescents and young adults. J Adolesc Health.

[49] Giovanelli A, Ozer EM, Dahl RE (2020). Leveraging technology to improve health in adolescence: a developmental science perspective. J Adolesc Health.

[50] Beaudry J, Consigli A, Clark C, Robinson KJ (2019). Getting ready for adult healthcare: designing a chatbot to coach adolescents with special health needs through the transitions of care. J Pediatr Nurs.

[51] Shah J, DePietro B, D'Adamo L, Firebaugh M-L, Laing O, Fowler LA, Smolar L, Sadeh-Sharvit S, Taylor CB, Wilfley DE, Fitzsimmons-Craft EE (2022). Development and usability testing of a chatbot to promote mental health services use among individuals with eating disorders following screening. Int J Eat Disord.

[52] Vigilante VA, Hossain J, Wysocki T, Sharif I (2015). Correlates of type and quantity of child communication during pediatric subspecialty encounters. Patient Educ Couns.

