# Supplementary material for: Acceptability of a Pain History Assessment and Education Chatbot (Dolores) Across Age Groups in Populations With Chronic Pain: Development and Pilot Testing
Source: JMIR Form Res. 2023 Oct 6;7:e47267. doi: 10.2196/47267 (PMC10589833; doi:10.2196/47267)
Supplement: Multimedia Appendix 2 [file formative_v7i1e47267_app2.pdf]

DOLORES GUIDE

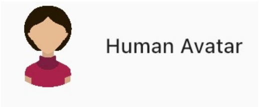

Tap to change to avatar

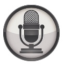

Click the microphone, begin speaking then click Dolores when you are finished speaking

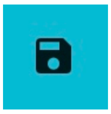

Click to save a PDF version of current text that can be provided to you at the conclusion of the interview

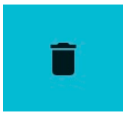

Click to delete a chat box you have created

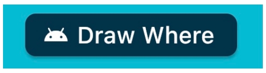

Click to draw

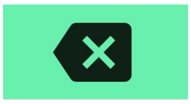

Click to undo last stroke

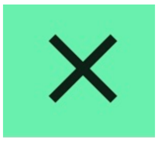

Click to delete the whole drawing

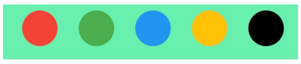

Choose a colour by selecting from suggested colours

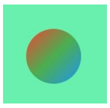

Choose to customise your colour

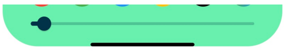

Edit the thickness of your pen by using the sliding scale

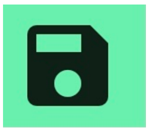

Click to save your drawing and continue to the next question

Ask Questions - If you have specific question regarding your pain or pain in general

Browse Topics - If you are unsure of where to start, this option will provide you with potential topics you may be interested in regarding pain.

Take a Quiz - This will provide you with a short quiz to test your knowledge about persistent pain

Random Topic - A random fact or detail will be provided to you regarding pain
