# Supplementary material for: Acceptability of a Pain History Assessment and Education Chatbot (Dolores) Across Age Groups in Populations With Chronic Pain: Development and Pilot Testing
Source: JMIR Form Res. 2023 Oct 6;7:e47267. doi: 10.2196/47267 (PMC10589833; doi:10.2196/47267)
Supplement: Multimedia Appendix 3 [file formative_v7i1e47267_app3.pdf]

Dolores Feedback Questionnaire

1) I enjoyed talking to Dolores.

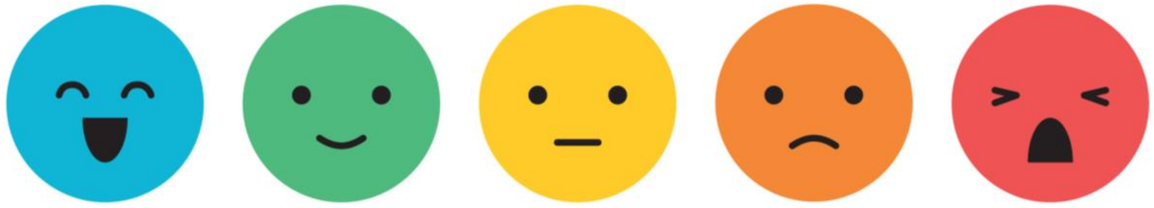

1. Strongly Agree 2. Agree 3. Neutral 4. Disagree 5. Strongly Disagree

2) Dolores helped me to understand more about my pain or my pain treatments.

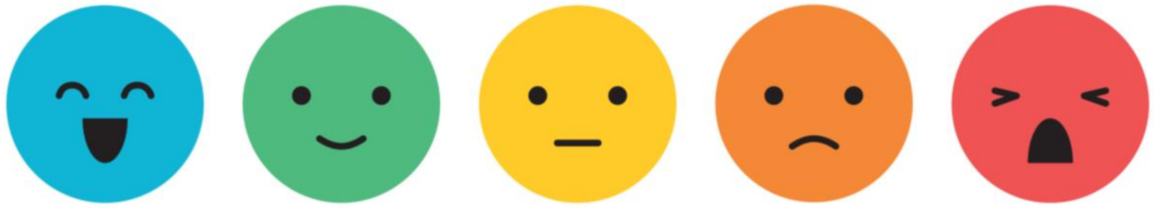

1. Strongly Agree 2. Agree 3. Neutral 4. Disagree 5. Strongly Disagree

3) It was easy to talk to Dolores.

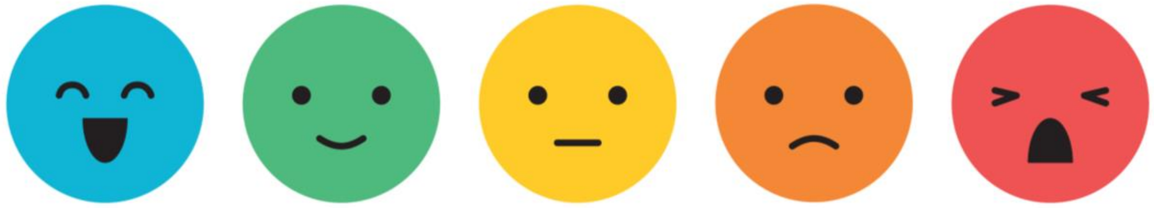

1. Strongly Agree 2. Agree 3. Neutral 4. Disagree 5. Strongly Disagree

4) Dolores understood what you I was asking/saying.

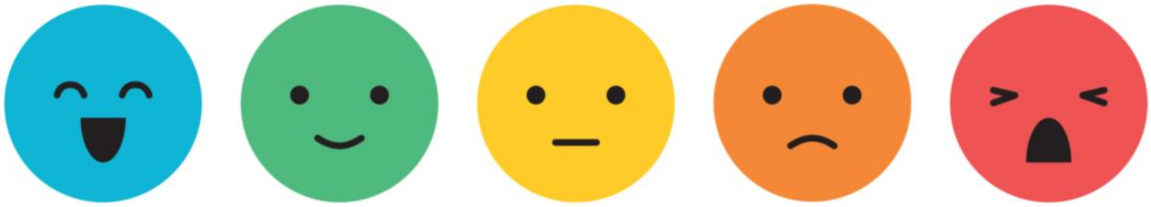

1. Strongly Agree 2. Agree 3. Neutral 4. Disagree 5. Strongly Disagree

5) Dolores was fast enough when responding back to me.

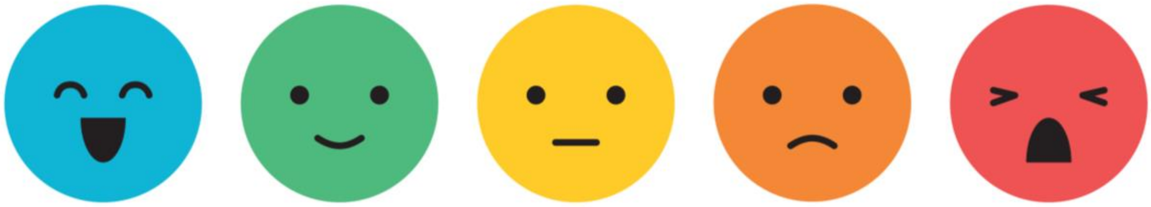

1. Strongly Agree 2. Agree 3. Neutral 4. Disagree 5. Strongly Disagree

6) I would talk to Dolores again.

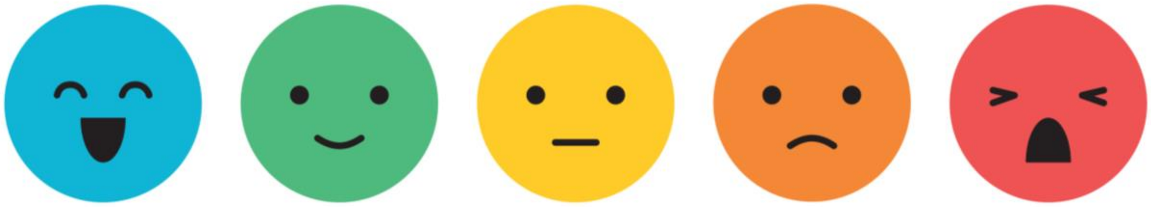

1. Strongly Agree 2. Agree 3. Neutral 4. Disagree 5. Strongly Disagree

7) **I liked the design of the Dolores App including graphics and layout.**

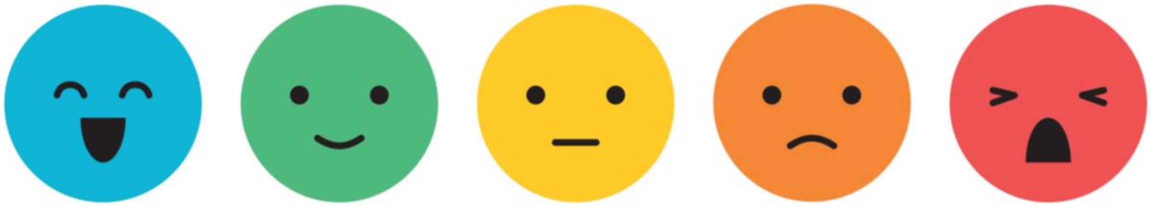

1. Strongly Agree 2. Agree 3. Neutral 4. Disagree 5. Strongly Disagree

8) I would recommend Dolores to others.

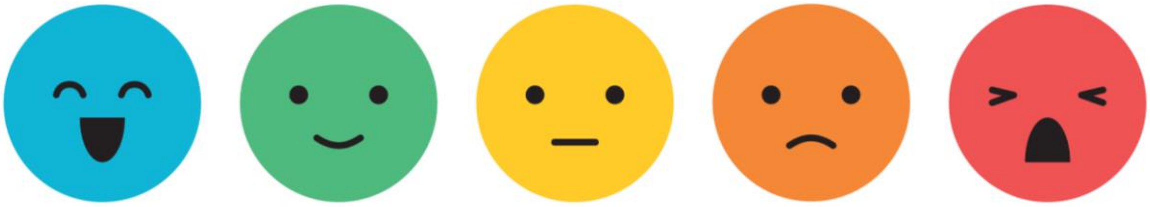

1. Strongly Agree 2. Agree 3. Neutral 4. Disagree 5. Strongly Disagree

9) What did you like the most about Dolores?

10) What did you like the least about Dolores?

11) What could make Dolores better?

12) Do you have any additional comments/concerns about the Dolores app?

Thank you for helping us improve the quality of mobile phone applications for people of all ages with persistent pain
